# Use of extracorporeal membrane oxygenation in postpartum patients with refractory shock or respiratory failure

**DOI:** 10.1038/s41598-020-80423-w

**Published:** 2021-01-13

**Authors:** Ryoung-Eun Ko, Chi Ryang Chung, Jeong Hoon Yang, Kyeongman Jeon, Gee Young Suh, Soo-young Oh, Suk-Joo Choi, Ji-Hyuk Yang, Kiick Sung, Yang Hyun Cho

**Affiliations:** 1 Department of Critical Care Medicine, Samsung Medical Center, Sungkyunkwan University School of Medicine, Seoul, Republic of Korea; 2 Department of Medicine, Samsung Medical Center, Sungkyunkwan University School of Medicine, Seoul, Republic of Korea; 3 Division of Cardiology, Department of Internal Medicine, Samsung Medical Center, Sungkyunkwan University School of Medicine, Seoul, Republic of Korea; 4 Division of Pulmonary and Critical Care Medicine, Department of Medicine, Samsung Medical Center, Sungkyunkwan University School of Medicine, Seoul, Republic of Korea; 5 Department of Obstetrics and Gynecology, Samsung Medical Center, Sungkyunkwan University School of Medicine, Seoul, Republic of Korea; 6 Department of Thoracic and Cardiovascular Surgery, Samsung Medical Center, Sungkyunkwan University of Medicine, 81 Irwon-ro, Gangnam-gu, Seoul, 06351 Republic of Korea

**Keywords:** Cardiac device therapy, Cardiovascular diseases, Reproductive disorders, Respiratory tract diseases

## Abstract

Although extracorporeal membrane oxygenation (ECMO) is increasingly utilized, only a limited level of experience has been reported in postpartum cardiopulmonary failure. Ten critically ill postpartum patients who received ECMO were included between January 2010 and December 2018 in this retrospective observational study. The main indication for ECMO support was peripartum cardiomyopathy (n = 5), followed by postpartum hemorrhage (n = 2). Nine patients initially received veno-arterial ECMO, and one patient received veno-venous ECMO. Major bleeding occurred in six patients. The median number of units of red blood cells (RBC) transfused during ECMO was 14.5 units (interquartile range 6.8–37.8 units), and most RBC transfusions occurred on the first day of ECMO. The survival-to-discharge rate was 80%. Compared to the survival outcomes in female patients of similar age who received ECMO, the survival outcomes were significantly better in the study population (56% versus 80%, *P* = 0.0004). Despite the high risk of major bleeding, ECMO for patients with postpartum cardiac or respiratory failure showed excellent survival outcomes. ECMO is feasible in these patients and can be carried out with good outcomes in an experienced centre.

## Introduction

Maternal mortality remains high globally, and it is estimated that 80% of peripartum deaths occur during the postpartum period within 2 weeks of delivery. Despite the remarkable enhancement in maternal healthcare over the past few decades, maternal mortality in the United States has increased from 10.3 per 100,000 live births in 1991 to 16.7 per 100,000 live births in 2016^1^. Besides, maternal mortality in Korea is still high^2^. The leading causes of maternal mortality are postpartum hemorrhage and cardiovascular conditions^3,4^. Therefore, early detection and management of postpartum cardiopulmonary failure is highly essential in reducing postpartum mortality.

Extracorporeal membrane oxygenation (ECMO) is increasingly used for refractory cardiac and respiratory failure^5,6^. Recent systematic reviews and analysis of Extracorporeal Life Support Organization Registry, however, revealed an overall maternal survival of 70–90% in paucity of peri-partum patients receiving ECMO support^7–10^. Although, the Mothers and Babies: Reducing Risk through Audits and Confidential Enquiries across the UK (MBRRACE-UL) report, which reported Confidential Enquiry into Maternal Deaths in the United Kingdom from 2009 to 2014, made recommendations for early ECMO referral to reduce maternal mortality for peripartum patients, limited guidelines for the use of ECMO in postpartum cardiopulmonary failure are available^11^. Moreover, little is known on the specific risks related to the use of ECMO during the postpartum period. Detailed information on coagulopathy in postpartum patients receiving ECMO is currently available, forcing clinicians to make various difficult bedside decisions. The current study attempts to provide our institutional experience in the management of postpartum patients receiving ECMO and describes pertinent indications and clinical outcomes.

## Results

### Patient characteristics at the time of ECMO

**Figure 1:**
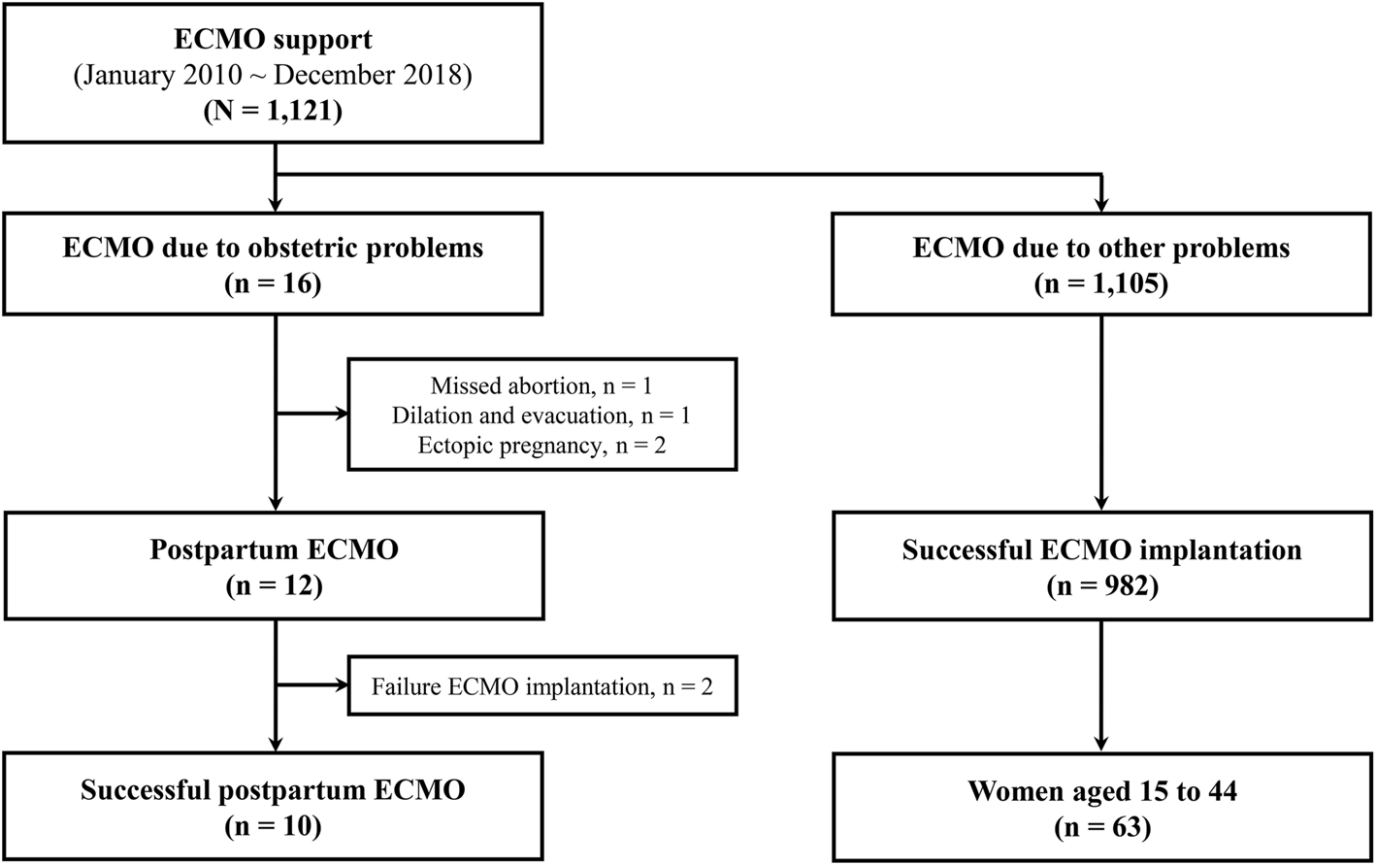
Flowchart of the study. *ECMO* extracorporeal membrane oxygenation.

During the study period, 1121 patients received ECMO support, and 16 patients received ECMO for obstetric problems. Among these patients, four patients were excluded from this study because they had abnormal-pregnancy-related problems. Of four patients, there were two patients with ectopic pregnancy, one with missed abortion, and one who had dilatation and evacuation. No patients received ECMO during pregnancy. After excluding patients with ECMO insertion failure (n = 2), 10 patients were included in the study (Fig. 1). The patient characteristics and mortality outcomes are summarized in Table 1. The median age was 33 years (IQR 30.2–35.0 years), and the median body mass index was 24.1 kg/m^2^ (IQR 22.7–26.0 kg/m^2^). All patients but one (90%) underwent cesarean delivery, and five of the nine patients underwent emergency cesarean delivery. The main reason for ECMO support was peripartum cardiomyopathy (CMP) (n = 5, 50%), followed by postpartum hemorrhage (n = 2, 20%). One patient had severe respiratory failure due to influenza B. Other causes were pulmonary embolism (n = 1, 10%) and refractory ventricular tachycardia (n = 1, 10%).

**Table 1:** Clinical characteristics and outcomes.

No.	Age, years	BMI at admission	Underlying disease	Gestational age at delivery, weeks	Parity	Emergent delivery	Reason for ECMO support	Insertion location	ECMO configuration	ICU mortality	Cause of death	CPC score at discharge
1	35	21.5	None	33	Multi	Yes	Peripartum CMP	ICU	V-A → V-V	Death	Sepsis	4
2	31	36.1	Asthma	36	Multi	No	PTE	Cath lab	V-A	Survival	−	1
3	35	19.5	None	31	Prim	Yes	ARDS	ICU	V-V	Survival	−	1
4	30	24.1	Dyslipidemia	30	Prim	Yes	Peripartum CMP	Cath lab	V-A	Survival	−	1
5	22	22.7	None	40	Multi	Yes	PPH, uterine atony	Cath lab	V-V	Survival	−	1
6	34	22.8	Breast cancer	36	Prim	No	Peripartum CMP	OR	E-LVAD	Survival	−	1
7*	30	24.2	None	N/A	Prim	No	PPH, placenta abruption	N/A	V-A	Survival	−	1
8*	41	26.6	None	N/A	Multi	No	Peripartum CMP	N/A	V-A	Survival	−	1
9*	32	30.2	None	N/A	Prim	Yes	Peripartum CMP	N/A	V-A	Survival	−	1
10	36	24.1	Asthma, h/o MVR	26	Prim	No	Prosthetic valve failure	ICU	V-A → V-V	Death	Hepatic failure	4

*BMI* body mass index, *MVR* mitral valve replacement, *ECMO* extracorporeal membrane oxygenator, *CMP* cardiomyopathy, *PTE* pulmonary thromboembolism, *ARDS* acute respiratory distress syndrome, *PPH* postpartum hemorrhage, *ICU* intensive care unit, *OR* operating room, *V-A* veno-arterial, *V-V* veno-venous, *E-LVAD* extracorporeal left ventricular assist device.

*Patients transferred after ECMO insertion.

### ECMO initiation and ICU management

**Figure 2:**
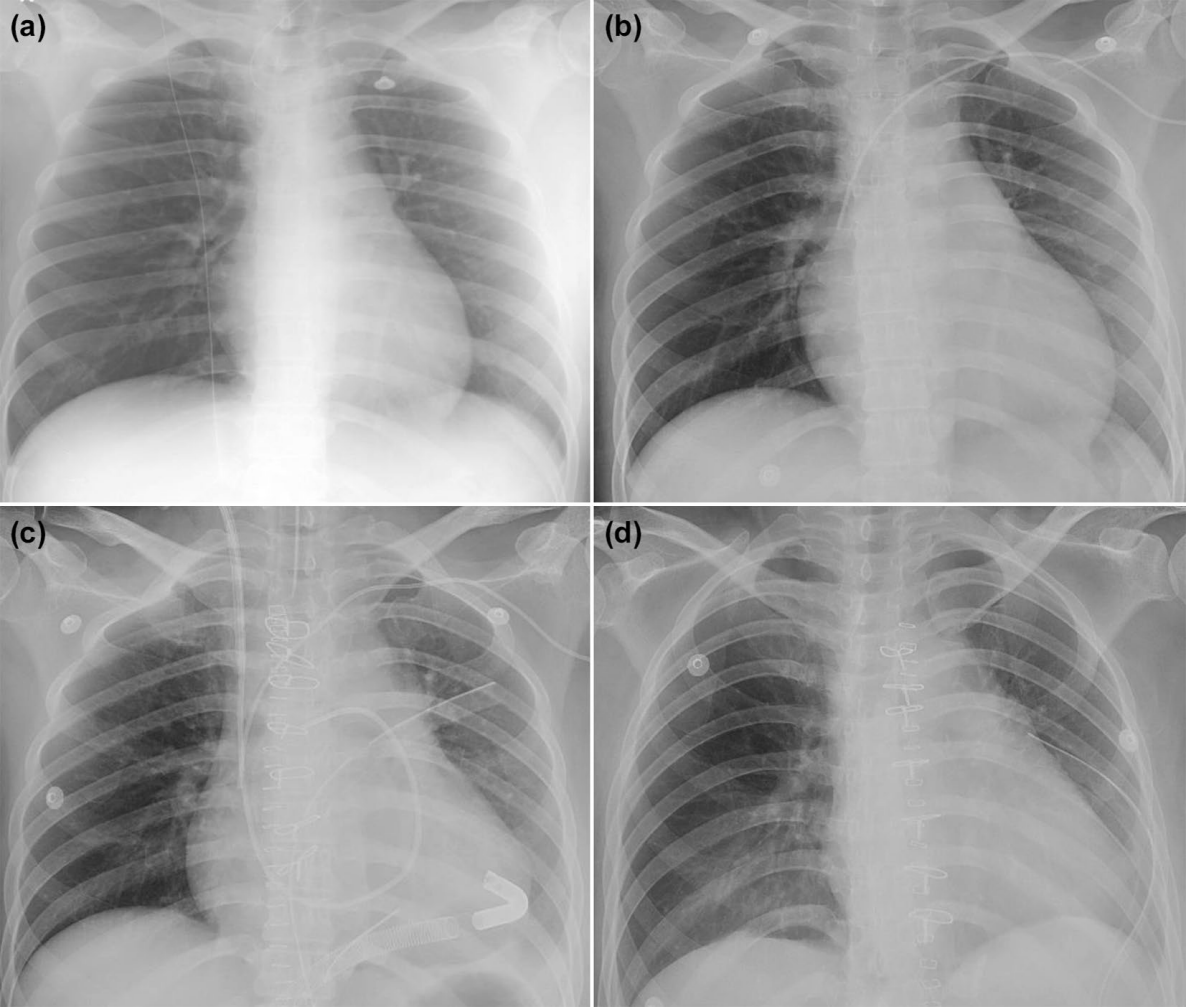
Representative postpartum patient who received ECMO. A 34-year-old pregnant woman with a fetus with a gestational age of 16 weeks was diagnosed with breast cancer stage IA, underwent right total mastectomy, and received adjuvant doxorubicin and cyclophosphamide chemotherapy. She delivered at a gestational age of 36 weeks and was discharged without significant symptoms (**a**). After 3 weeks, she presented to the emergency room with dyspnea and palpitations (**b**). An echocardiogram revealed decreased biventricular systolic function with an LVEF of approximately 25%. After 2 weeks of intensive care unit management with dobutamine and volume control, her symptoms did not improve. Emergency extracorporeal LVAD insertion was performed, and 8 days after extracorporeal LVAD insertion (**c**), she underwent heart transplantation (**d**). *ECMO* extracorporeal membrane oxygenation, *LVEF* left ventricle ejection fraction, *LVAD* left ventricular assist device.

All patients received ECMO after delivery. The locations of ECMO insertion were the cardiac catheterization laboratory (n = 3, 30%), ICU (n = 3, 30%), operating room (n = 1, 10%), and other hospitals (n = 3, 30%) (Table 1). The median time from delivery to ECMO insertion in in-hospital patients receiving ECMO was 37.0 h (IQR 24.5–163.3 h). Nine patients (90%) initially received V-A ECMO. One patient (10%) received veno-venous (V-V) ECMO to manage severe pulmonary edema caused by a massive transfusion for hemorrhagic shock. Among the patients who received V-A ECMO, one patient underwent extracorporeal left ventricular assist device insertion as a bridge to heart transplantation. The detailed course of the patient undergoing bridge to heart transplantation is described in Fig. 2.

ICU management and ECMO support are described in detail in Supplementary Table 1. All patients were intubated and mechanically ventilated before ECMO insertion. Nine patients (90%) required at least one vasopressor on the day of ECMO insertion. Seven patients (70%) received continuous renal replacement therapy. The drain and return cannula sizes varied from 19 to 28 Fr and from 15 to 24 Fr, respectively. The percutaneous Seldinger’s technique was used in nine patients (90%). The median initial ECMO flow rate was 3.4 L/min (IQR 3.1–3.6 L/min). Three patients (30%) underwent left heart venting, and two patients (20%) underwent distal limb perfusion.

### Complications and clinical outcomes

**Figure 3:**
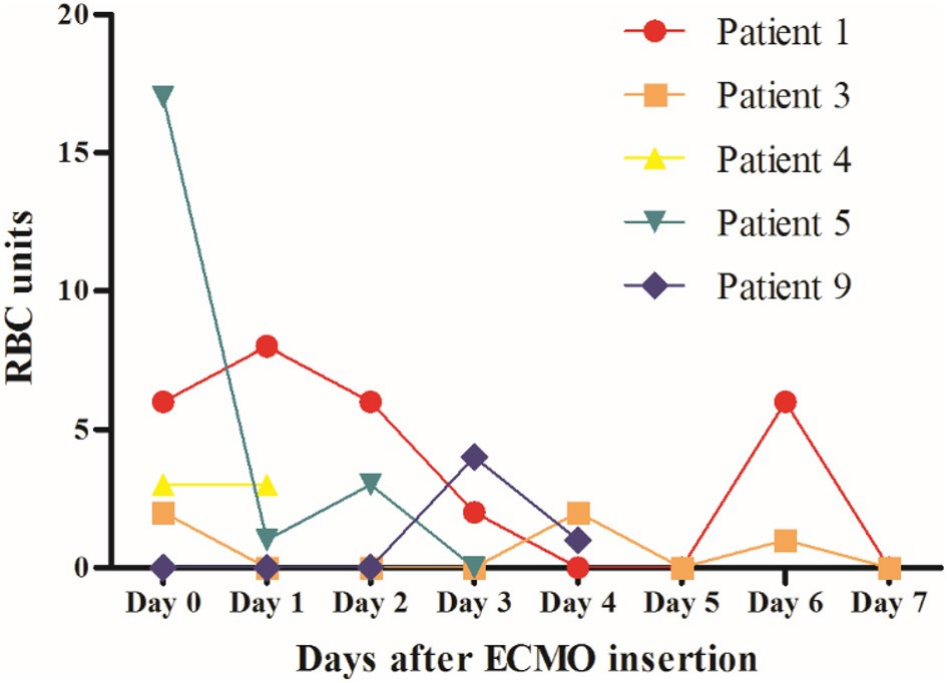
Transfusions in patients who underwent an emergency cesarean delivery. Although blood product requirement was high during early period of extracorporeal membrane oxygenation, it was decreased afterwards. *RBC* red blood cell, *ECMO* extracorporeal membrane oxygenation.

All patients except one (90%) received therapeutic anticoagulation with unfractionated heparin. Detailed information on coagulopathy, bleeding and transfusion are described in Table 2. One patient did not receive anticoagulation due to large abdominal wall hematoma (9 × 3 cm) in the rectus muscle at the surgical incision site for caesarian section. Major bleeding occurred in six patients (60%). The sources of bleeding were the cannula insertion site in two patients, pulmonary hemorrhage in two, vaginal bleeding in two, and the abdominal wall in one. The median number of units of RBCs transfused during ECMO was 14.5 units (IQR 6.8–37.8 units), and the median number of units of RBCs transfused within 24 h after ECMO was 2.5 units (IQR 0.5–6.8 units). Figure 3 shows the RBC transfusions within seven days in five patients who underwent emergency cesarean delivery and ECMO insertion. Most RBC transfusions occurred on the first day of ECMO. There were no thromboembolic events during ECMO support.

**Table 2:** Bleeding results during ECMO.

No.	Anticoagulation	Median aPTT, s (IQR)	Major bleeding	Delivery-related bleeding	Total RBC units	RBC transfusions within 24 h	RBC units per day
1	Heparin	63.4 (58.6–73.8)	Lung hemorrhage	No	43	6	2
2	Heparin	59.5 (46.9–94.0)	Catheter site bleeding	No	85	18	64
3	Heparin	60.0 (53.9–67.2)	Catheter site bleeding	No	14	2	1
4	Heparin	120.1 (94.3–156.7)	None	No	6	3	4
5	Heparin	45.8 (40.3–80.1)	Postpartum bleeding	No	22	18	9
6	Heparin	56.1 (52.1–65.1)	None	No	15	0	2
7	Heparin	63.3 (53.3–70.6)	None	No	9	2	1
8	None	58.3 (57.0–59.6)	Muscle hematoma	Subfascial hematoma	6	0	1
9	Heparin	58.4 (55.1–61.5)	None	No	5	0	1
10	Heparin	49.4 (45.1–53.9)	Lung hemorrhage	No	45	7	4

*ECMO* extracorporeal membrane oxygenation, *aPTT* activated partial thromboplastin time, *IQR* interquartile range, *RBC* red blood cell.

**Figure 4:**
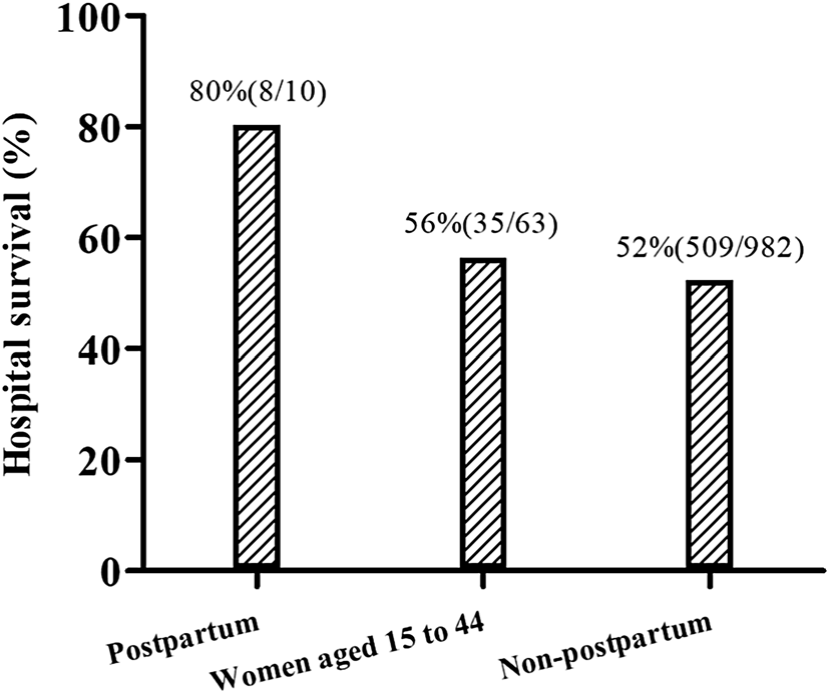
Hospital survival of patients: the survival of postpartum patients was significantly better than that of female patients with age between 15 to 44.

Eight (80%) patients were successfully weaned from ECMO and discharged from the hospital (Table 1). No survivors had major neurologic deficits. The cause of death in two (20%) patients was sepsis and liver failure, respectively. Compared to women aged 15–44 years who received ECMO at our institution, the study population showed better hospital survival rate (56% versus 80%, *P* = 0.0004) (Fig. 4). The median ECMO duration was 217.0 h (IQR 68.0–300.7 h), and the median mechanical ventilation support duration was 8.0 days (IQR 5.2–41.2 days). The median length of ICU and hospital stay was 19.0 days (IQR 14.5–23.0 days) and 28.0 days (IQR 20.0–38.5 days), respectively. Limb ischemia was noted in one patient. This condition developed in conjunction with compartment syndrome due to catheter insertion-related iliofemoral artery thrombosis. The patient was able to ambulate with a brace upon hospital discharge. No serious technical difficulties during ECMO support, such as accidental decannulation, disconnection, oxygenator failure, hemolysis, air emboli, and other circuit complications, were noted in this patient cohort.

## Discussion

In the present study, we described 10 postpartum patients who underwent ECMO. The major findings of this study were as follows: (1) Although bleeding complications and transfusion requirements were high, the rates of these events were similar to those in previous studies^12–14^. (2) ECMO for postpartum cardiopulmonary failure was effective and led to significantly better hospital survival. (3) ECMO-related complications, except bleeding in the first day of ECMO insertion, were infrequent in postpartum patients receiving ECMO with multidisciplinary ECMO team management.

Cardiopulmonary failure often occurs in both the prenatal and postpartum period. Accordingly, alert and active management must be implemented to cope with cardiopulmonary collapse. The use of post-partum ECMO has been documented in a few patients with cardiomyopathy and other infective etiologies^15–17^. The reason for the lower frequency of ECMO use in postpartum patients may be due to insufficient clinical experience in ECMO use in this patient population. In this study, we described various postpartum problems, including peripartum CMP and postpartum hemorrhage, and our findings confirmed that ECMO use is feasible for postpartum patients with favorable outcomes. As seen with previous reports, we also demonstrated increased survival rate up to 80%. This might also be related to patient characteristics: these patients are relatively young, constantly monitored when they are ante, intra and post-partum that facilitates early detection of pathologies and their physiological reserves are already tested during pregnancy.

It is estimated that 60% of pregnancy related heart failure happens in the post-partum period, leading to a fourfold increase in mortality^18^. Hypertension and cardiomyopathy are the most common etiologies. Peripartum CMP is a potentially life-threatening pregnancy-associated disease marked by left ventricular dysfunction and heart failure^17,19^. The ELSO registry report survival rates of 64% in patients with peripartum CMP. Other studies that described ECMO use in peripartum CMP are limited mainly to single center case reports^20–24^. We encountered five patients with peripartum CMP who received ECMO during the postpartum period. Among these five patients, four patients received V-A ECMO as a bridge to recovery, and one patient underwent extracorporeal left ventricular assist device insertion as a bridge to transplantation. One patient receiving V-A ECMO as a bridge to recovery died due to sepsis, but the others survived. These findings suggest that V-A ECMO in patients with peripartum CMP should be considered for refractory cardiac or respiratory failure.

Several studies have highlighted use of ECMO in mixed prenatal and postpartum patients^25–28^. One of the largest series covered 12 pregnant or postpartum patients who underwent ECMO for severe acute respiratory distress syndrome (ARDS) during the 2009 H1N1 pandemic^25^. Among these 12 patients, only 5 postpartum patients with severe ARDS received ECMO (2V-A ECMO to V-V ECMO and 3V-V ECMO). Several meta-regression analysis also showed the efficacy of ECMO for pregnant and post-partum patients with cardiopulmonary failure. Zhang et al. analyzed 9 observational studies of peri-partum patients supported with ECMO showed acceptable pooled prevalence of bleeding complications and fetal survivals in addtion to good maternal outcomes^9^. Another review analyzed 90 case reports of pregnant and postpartum patients and showed excellent survival rate up to 91.5% of postpartum patients who received ECMO^7^.

The most significant limitations affecting ECMO in postpartum patients may be related to concerns about hypercoagulability and bleeding. The most common problem associated with ECMO is bleeding^29^. Bleeding events have been estimated to occur in 30–60% of patients, depending on the type of extracorporeal life support and indication for use^12,30,31^. We also observed an increase in the proportion of patients with major bleeding episodes, which reached approximately 60% in our cohort. However, increased major bleeding did not affect the patient mortality rate. Furthermore, two patients who received ECMO had postpartum hemorrhage but survived without significant complications. Thrombotic complications were not observed in this study.

There are several potential limitations to our study that should be acknowledged. First, this retrospective study was limited by the small number of patients and associated with inherent shortcomings of the study design, including the lack of randomization of treatment with ECMO or standard care. Second, this study was conducted at a single center with a specialized ECMO team, which thus limits the generalizability of our findings to other institutions or patient populations.

## Conclusion

Despite the high incidence of major bleeding, ECMO for postpartum cardiac or respiratory failure showed excellent survival outcomes. ECMO is feasible in these patients and can be carried out with good outcomes in an experienced centre.

## Methods

### Study design and population

This was a retrospective, single-center, observational study of adult patients who received ECMO during the postpartum period at Samsung Medical Center (a 1979-bed, university-affiliated, tertiary referral hospital in Seoul, South Korea) between January 2010 and December 2018. All consecutive patients who received ECMO were screened for inclusion in this study. Patients who had obstetric problems within 6 weeks after delivery that mandated ECMO support were included in this study. Patients who had abnormal-pregnancy-related problems, received ECMO during pregnancy, or had ECMO insertion failure were excluded. ECMO insertion failure was defined as a condition in which meaningful ECMO flow is not achieved after ECMO pump-on.

This study was approved by the Institutional Review Board of Samsung Medical Center (IRB no. 2019-10-116). Informed consent was waived because of the retrospective observational nature of the study. Patient information was anonymized and de-identified prior to analysis. All methods were performed in accordance with the relevant guidelines and regulations.

### ECMO management

Details of our ECMO management were described in previous reports^32–34^. Patients with profound cardiopulmonary failure were considered candidates for ECMO, and ECMO initiation was determined after multidisciplinary discussion by intensivists, cardiologists, cardiovascular surgeons, and obstetricians^32^. We have also accepted patient on ECMO from other hospitals. We send specialized ECMO transport team for safety^35^. Our multidisciplinary ECMO team, which includes intensivists, pulmonologists, and cardiothoracic surgeons, conducted daily rounds and assessed the circuit status, development of ECMO-associated complications, and possibility of weaning^32,34^. Arterial, central venous, and/or pulmonary artery catheters were utilized for continuous hemodynamic monitoring^32^. The pump blood flow rate was initially adjusted to sustain a mean arterial pressure of 60–90 mmHg and then targeted for adequate tissue perfusion in veno-arterial (V-A) ECMO^32^. Intravenous fluids, blood products, vasopressors, inotropes, or vasodilators were infused as needed. An ECMO weaning trial was considered when patients were hemodynamically stable with or without a low level of pharmacologic support and with adequate native lung oxygenation capacity^32^. When major bleeding happened, the heparin was stopped, and the use of protamine or blood products for reversal of heparin was determined by consultation with ECMO specialists. We also reversed coagulopathies and performed interventional or surgical hemostasis, whenever possible. Then, heparin was resumed when bleeding related-hemodynamic impairment was resolved and a lower target of 120–150 s for activated clotting time was aimed for.

### Data collection

Trained study coordinators prospectively collected the clinical and laboratory data using a standardized case report form. The following information was obtained from the medical records: general demographic information, gestational age at delivery, presence of selected predefined underlying diseases, delivery type, reason for ECMO support, need for mechanical ventilation support, need for renal replacement therapy, need for vasopressor support, ECMO insertion site, ECMO type, and technical details of ECMO, including vascular cannulation, initial flow, venting, and distal perfusion. We also reviewed anticoagulant uses, median activated partial thromboplastin time, major bleeding events, and blood transfusions. A major bleeding complication was defined as a bleeding event that required transfusion of ≥ 2 units of packed red blood cells (RBCs). Thrombotic complications were defined as intracranial infarction, limb ischemia, pulmonary emboli, or intracardiac thrombi^36^. Outcomes, such as the length of the intensive care unit (ICU) and hospital stays, ECMO duration, mechanical ventilation duration, limb ischemia, neurologic outcomes, and ICU mortality, were documented.

### Statistical analysis

The data were summarized using descriptive statistics. The median and interquartile range (IQR; 25th and 75th percentiles) were calculated for continuous variables, while the frequency and percentage were calculated for categorical variables. No assumptions were made regarding missing data and adjusted proportions to the number of patients with available data. All data analyses were performed using R Statistical Software (version 3.2.5; R Foundation for Statistical Computing, Vienna, Austria).

### Ethics approval and consent to participate

The Institutional Review Board of Samsung Medical Center approved this study and waived the requirement for informed consent because of the observational nature of the study.

## Supplementary Information

Supplementary Table 1.

## Data Availability

The data that support the findings of this study are available on request from the corresponding author. The data are not publicly available due to privacy or ethical restrictions.

## References

[1] Fridkin SK, Welbel SF, Weinstein RA (1997). Magnitude and prevention of nosocomial infections in the intensive care unit. Infect. Dis. Clin. N. Am..

[2] Park HS, Kwon H (2016). Analysis of the causes and trends of maternal mortality in Korea: 2009–2014. Korean J. Perinatol..

[3] Nour NM (2008). An introduction to maternal mortality. Rev. Obstet. Gynecol..

[4] Creanga AA, Syverson C, Seed K, Callaghan WM (2017). Pregnancy-related mortality in the United States, 2011–2013. Obstet. Gynecol..

[5] Hsu CP (2015). Extracorporeal membrane oxygenation use, expenditure, and outcomes in Taiwan from 2000 to 2010. J. Epidemiol..

[6] Tay CK (2019). Extracorporeal membrane oxygenation in Korea - Trends and impact of hospital volume on outcome: Analysis of national insurance data 2009–2014. J. Crit. Care.

[7] Ong J, Zhang JJY, Lorusso R, MacLaren G, Ramanathan K (2020). Extracorporeal membrane oxygenation in pregnancy and the postpartum period: A systematic review of case reports. Int. J. Obstet. Anesth..

[8] Ramanathan K (2020). Extracorporeal membrane oxygenation in pregnancy: An analysis of the extracorporeal life support organization registry. Crit. Care Med..

[9] Zhang JJY (2019). Extracorporeal membrane oxygenation in pregnant and postpartum women: A systematic review and meta-regression analysis. J. Intensive Care Med..

[10] Olson TL (2020). Extracorporeal membrane oxygenation in peripartum cardiomyopathy: A review of the ELSO registry. Int. J. Cardiol..

[11] Kurinczuk JJ (2014). Experiences with maternal and perinatal death reviews in the UK–the MBRRACE-UK programme. BJOG.

[12] Cheng R (2014). Complications of extracorporeal membrane oxygenation for treatment of cardiogenic shock and cardiac arrest: a meta-analysis of 1866 adult patients. Ann. Thorac. Surg..

[13] Fitzgerald RK, Davis AT, Hanson SJ, Related Institution PICU Focus Group Investigators (2015). Multicenter analysis of the factors associated with unplanned extubation in the PICU. Pediatr. Crit. Care Med..

[14] Agerstrand C (2016). Extracorporeal membrane oxygenation for cardiopulmonary failure during pregnancy and postpartum. Ann. Thorac. Surg..

[15] Hemorrhage P (2017). Committee on practice bulletins-obstetrics. Practice bulletin no. 183. Obstet. Gynecol..

[16] World Health Organization. *WHO Guidelines for the Management of Postpartum Haemorrhage and Retained Placenta*. World Health Organization. https://apps.who.int/iris/handle/10665/44171 (2009). 23844453

[17] Bauersachs J (2019). Pathophysiology, diagnosis and management of peripartum cardiomyopathy: A position statement from the Heart Failure Association of the European Society of Cardiology Study Group on peripartum cardiomyopathy. Eur. J. Heart Fail..

[18] Mogos MF (2018). Heart failure in pregnant women: A concern across the pregnancy continuum. Circ. Heart Fail.

[19] Pearson GD (2000). Peripartum cardiomyopathy: National heart, lung, and blood institute and office of rare diseases (National Institutes of Health) workshop recommendations and review. JAMA.

[20] Bouabdallaoui N, Mastroianni C, Revelli L, Demondion P, Lebreton G (2015). Predelivery extracorporeal membrane oxygenation in a life-threatening peripartum cardiomyopathy: Save both mother and child. Am. J. Emerg. Med..

[21] Kim HY (2014). Anesthetic experience using extracorporeal membrane oxygenation for cesarean section in the patient with peripartum cardiomyopathy: a case report. Korean J. Anesthesiol..

[22] Mikami T, Kamiunten H (2018). Emergent caesarean section under mechanical circulatory support for acute severe peripartum cardiomyopathy. J. Cardiol. Cases.

[23] Ohira S (2018). A left ventricular assist device for a patient with peripartum cardiomyopathy. J. Surg. Case Rep..

[24] Palanzo DA (2009). Successful treatment of peripartum cardiomyopathy with extracorporeal membrane oxygenation. Perfusion.

[25] Nair P (2011). Extracorporeal membrane oxygenation for severe ARDS in pregnant and postpartum women during the 2009 H1N1 pandemic. Intensive Care Med..

[26] Itagaki T (2014). Successful use of extracorporeal membrane oxygenation in the reversal of cardiorespiratory failure induced by atonic uterine bleeding: A case report. J. Med. Case Rep..

[27] Shen HP, Chang WC, Yeh LS, Ho M (2009). Amniotic fluid embolism treated with emergency extracorporeal membrane oxygenation: A case report. J. Reprod. Med..

[28] Jo YY, Park S, Choi YS (2011). Extracorporeal membrane oxygenation in a patient with stress-induced cardiomyopathy after caesarean section. Anaesth. Intensive Care.

[29] Thomas J, Kostousov V, Teruya J (2018). Bleeding and thrombotic complications in the use of extracorporeal membrane oxygenation. Semin. Thromb. Hemost..

[30] Schmidt M (2015). Mechanical ventilation management during extracorporeal membrane oxygenation for acute respiratory distress syndrome: A retrospective international multicenter study. Crit. Care Med..

[31] Kanji HD (2010). Peripheral versus central cannulation for extracorporeal membrane oxygenation: A comparison of limb ischemia and transfusion requirements. Thorac. Cardiovasc. Surg..

[32] Na SJ (2019). Left heart decompression at venoarterial extracorporeal membrane oxygenation initiation in cardiogenic shock: Prophylactic versus therapeutic strategy. J. Thorac. Dis..

[33] Park C (2019). Community versus hospital-acquired pneumonia in patients requiring extracorporeal membrane oxygenation. Ther. Adv. Respir. Dis..

[34] Na SJ (2018). The effect of multidisciplinary extracorporeal membrane oxygenation team on clinical outcomes in patients with severe acute respiratory failure. Ann. Intensive Care.

[35] Lee H (2020). Outcomes of transported and in-house patients on extracorporeal life support: A propensity score-matching study. Eur. J. Cardiothorac. Surg..

[36] Dalton HJ (2017). Factors associated with bleeding and thrombosis in children receiving extracorporeal membrane oxygenation. Am. J. Respir. Crit. Care Med..

