# Supplementary material for: Use of extracorporeal membrane oxygenation in postpartum patients with refractory shock or respiratory failure
Source: Sci Rep. 2021 Jan 13;11:887. doi: 10.1038/s41598-020-80423-w (PMC7806987; doi:10.1038/s41598-020-80423-w)
Supplement: Supplementary file 1 — Supplementary Table 1. [file 41598_2020_80423_MOESM1_ESM.docx]

**Supplementary Table 1.** ICU management and technical details of ECMO support.

| No. | MV support | CRRT | Vasopressor | Size of drain cannula, mm | Size of return cannula, mm | Initial flow, L/min | Venting | Distal perfusion |
| --- | --- | --- | --- | --- | --- | --- | --- | --- |
| 1 | Yes | Yes | Yes | 22 | 15 | 3.1 | No | No |
| 2 | Yes | Yes | Yes | 22 | 16 | 4 | Percutaneous | No |
| 3 | Yes | No | Yes | 28 | 18 | 3.6 | No | No |
| 4 | Yes | No | Yes | 22 | 16 | 3.2 | No | Yes |
| 5 | Yes | Yes | Yes | 22 | 20 | 3.6 | No | No |
| 6 | Yes | No | Yes | 24 | 24 | 3.4 | No | No |
| 7^*^ | Yes | Yes | No | 21 | 17 | 4.2 | No | Yes |
| 8^*^ | Yes | Yes | Yes | 19 | 15 | N/A | Surgical | No |
| 9^*^ | Yes | Yes | Yes | 19 | 15 | 2.7 | No | No |
| 10 | Yes | Yes | Yes | 22 | 15 | 2.5 | Surgical | No |

ICU, intensive care unit; ECMO, extracorporeal membrane oxygenator; MV, mechanical ventilation; CRRT, continuous renal replacement therapy.

^*^Patients transferred after ECMO insertion.
